# Learning multiple sclerosis immunopathogenesis from anti-CD20 therapy

**DOI:** 10.1073/pnas.2221544120

**Published:** 2023-01-31

**Authors:** Michael Heming, Heinz Wiendl

**Affiliations:** ^a^Department of Neurology with Institute of Translational Neurology, University of Münster, 48149 Münster, Germany

**Fig. 1.**
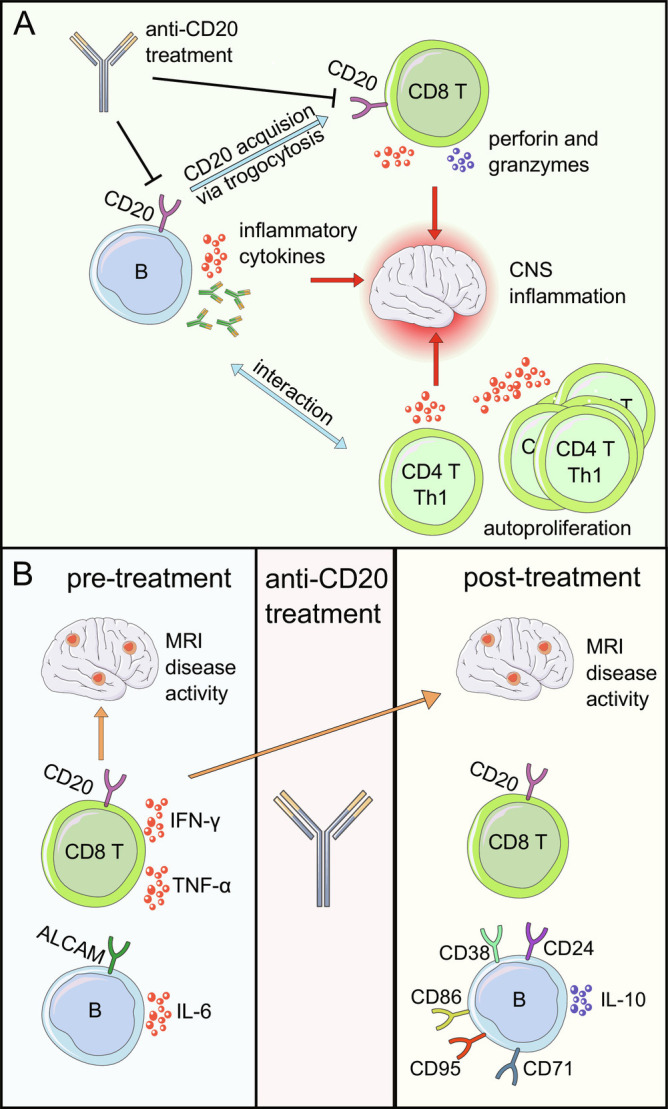
Multifaceted effects of anti-CD20 treatment in MS. (*A*) Shinoda et al. (19) show that CD20^dim^ T cells, particularly CD20^dim^ CD8^+^ T cells, correlate negatively with MRI disease activity before treatment and may also predict early MRI disease activity after treatment. Repopulating CD20^dim^ CD8^+^ T cells exhibit a less proinflammatory phenotype following anti-CD20 treatment. Reconstituting B cells are mainly transitional B cells (CD24^high^ and CD38^high^) with an anti-inflammatory (reduced IL-6 expression and increased IL-10 expression) and activated (CD86, CD95, and CD71) phenotype and reduced CNS homing properties (reduced ALCAM expression). (*B*) Proinflammatory B cells produce antibodies and proinflammatory cytokines that contribute to the CNS-directed autoimmune response. Additionally, they interact with CD4^+^ Th1 cells, which leads to an autoproliferation of Th1 cells. Proinflammatory CD20^dim^ CD8^+^ T cells acquire CD20 from B cells via trogocytosis and trigger relapses in MS. Anti-CD20 treatment depletes B cells, thereby reducing both proinflammatory B cells and T cell autoproliferation, and proinflammatory CD20^dim^ CD8^+^ T cells. The figure was partly created using Servier Medical Art, provided by Servier, licensed under a Create Commons Attribution 3.0 unported license.

Multiple sclerosis (MS) is an inflammatory neurodegenerative disorder with putative autoimmune origin. The disease starts earlier than current diagnostic criteria can detect and affects the entire central nervous system, and not only white matter as its original term—inflammatory demyelinating disorder—suggests. It is characterized by a very heterogeneous disease course represented by relapse-associated neurological worsening but also relapse-independent disability accrual (1). The advent of specific immune therapies, with now 20 FDA-approved therapies, represents a crucial part of the tremendous advancement the modality of this disease has made over the last 20 y. Alongside significantly changing the natural disease course and fate of individuals affected, a stunning gain of knowledge in the field was generated applying a novel paradigm exemplifying reverse translational medicine: learning immunopathogenesis via therapeutic intervention (2). Prominent examples include insights gained from leukocyte trafficking interference with natalizumab (preventing migration of leukocytes into the central nervous system (CNS) via VLA-4 neutralization) (3, 4), cladribine (reduction of B and T cells) (5), alemtuzumab (depletion of CD52^+^ immune cells) (6, 7), and rituximab/ocrelizumab/ofatumumab/ublituximab (depletion of CD20^+^ immune cells) (8–11). Using samples from different compartments (mainly peripheral blood, cerebrospinal fluid, and CNS tissue) helped to unravel mechanisms of relapsing and nonrelapsing MS biology. Since effects of applied immune therapies are mainly directed outside the central nervous system, the peripheral immune system is central for correcting immune-regulatory network disturbances. The strong dependence on T cells in MS animal models (experimental autoimmune encephalomyelitis, EAE) and the absence of antibodies in MS have long driven the assumption of MS being orchestrated by T helper cells (12), making those the most plausible therapeutic targets. But anti-CD20 therapies have been a game changer in the MS field. CD20-depleting antibodies effectively reduce relapses and progression in MS (8–11). Several studies show that CD20-expressing B cells are central to MS disease biology beyond producing antibodies (13). However, anti-CD20 therapy also affects T cells, both directly and indirectly (Fig. 1*A*). Indirectly, it reduces T cell autoproliferation mediated by memory B cells (14), among others. Directly, anti-CD20 treatment depletes CD20^dim^ T cells (15). While CD20 is primarily expressed on B cells at different stages excluding pro-B cells, plasma cells, and most plasmablasts, a small subset of T cells expresses CD20 (15). CD20^dim^ T cells have a proinflammatory phenotype and correlate with MS disease severity (16), and myelin-specific CD8^+^ T cells show an increased proportion of CD20 expression in MS (17). Recently, it was revealed that T cells can acquire CD20 from B cells via a mechanism called trogocytosis (18). Adoptive transfer of CD20-expressing T cells into EAE models deteriorated the disease severity (18). Impressively, the specific depletion of CD20-expressing T cells (by transferring myelin oligodendrocyte glycop (MOG)-primed WT T cells into CD20 KO mice) improves EAE independent of B cells (18), making CD20-expressing T cells an additional “culprit” in MS immunopathogenesis.

Shinoda et al. (19) started another reverse translation project with the observation that a small proportion of MS patients experience early disease in the first 3 to 6 mo after the initial anti-CD20 dose (9, 10). The team, spearheaded by Amit Bar-Or, investigated blood samples from two well-characterized MS cohorts, who started a therapy with the anti-CD20 treatment ocrelizumab. In contrast to most previous studies, which focused on either T or B cells, the authors provide an in vivo study of anti-CD20 treatment on both T and B cells and their interactions, thereby acknowledging that MS is an immune-regulatory network disorder, not a disease of a single-cell subset. The entire discovery cohort and the majority of the validation cohort did not receive disease-modifying therapy (DMT) previously, making them the best possible, least “contaminated” immunological cohort. Strikingly, they revealed a negative correlation between pretreatment MRI disease activity (numbers of gadolinium lesions), and CD20^dim^ T cells, particularly CD20^dim^ CD8^+^ T cells, i.e., patients with lower numbers of CD20^dim^ T cells showed more MRI lesions before treatment (Fig. 1*B*). Pathophysiologically, this suggests, as the authors speculate, that CD20^dim^ T cells leave the circulation and may enter CNS compartments, where they contribute to the immune response–triggering relapses. This hypothesis is supported by a previous study (16), which showed a correlation between CD20^dim^ T cells in the CSF of MS patients and their disease activity.

In accordance with previous studies (15, 17, 18), Shinoda et al. (19) observed a small population of CD20^dim^ T cells before treatment (3.1% of CD4^+^, 13.4% of CD8^+^ T cells), which was strongly reduced after anti-CD20 treatment. Interestingly, the decrease of CD20^dim^ CD8^+^ T cells accounted for 38% of the reduction of T effector memory cells (Tem), while similar trends between CD20^dim^ CD4^+^ T cells and CD4^+^ T cells were less pronounced. Reemerging CD20^dim^ CD8^+^ T cells contained fewer Tem and displayed a less proinflammatory phenotype (reduced expression of TNF-α and IFN-γ) in comparison to pretreatment (Fig. 1*B*). Reconstitution of CD20^dim^ T cells was not associated with disease activity, suggesting that CD20^dim^ T cells prior to anti-CD20 therapy and not the repopulating CD20^dim^ T cells contribute to relapse biology.

Following the initial dose of anti-CD20 treatment, B cells were nearly completely depleted 2 to 4 mo later as expected. A limited B cell reconstitution was observed 6 to 7 mo after initial anti-CD20 therapy. In line with previous research (20), repopulating B cells were mainly transitional B cells (CD24^high^ CD38^high^) with a proliferative (Ki67) and activated (CD86, CD95, and CD71) phenotype (Fig. 1*B*). Reconstituting B cells also showed an anti-inflammatory phenotype (increased IL-10, reduced IL-6 expression) with reduced CNS homing properties (reduced ALCAM expression). Interestingly, there was no correlation found between the presence of early repopulating B cells and disease activity.

Personalization of neurological immune therapy by predicting individual treatment responses becomes more important as the arsenal of available DMTs for MS rapidly grows. While this personalized approach is widely used in clinical oncology, its use in neuroimmunology lags behind. Larger, hypothesis-free approaches in well-curated cohorts with high-quality samples (ideally from distinct compartments and longitudinal follow-up) are needed to establish biomarkers to support diagnosis, prognosis, or disease monitoring. Promising examples of immune signatures using deep immune phenotyping approaches have been carried forward recently: Immunophenotyping of blood by mass cytometry identified a memory T helper cell population that correlated with treatment response of dimethyl fumarate in MS patients (21). While high-dimensionality blood immune flow cytometry profiles following CD52-depletion with alemtuzumab did not correlate with the risk of secondary autoimmune, T cell receptor analysis exhibited hyperexpanded T cell clones prior to treatment in the group that developed secondary autoimmunity (7).

Shinoda et al. (19) found that CD20^dim^ T cells and CD20^dim^ CD8^+^ T cells were predictive of early MRI disease activity after anti-CD20 treatment, in contrast to all other immune cell populations investigated, such as B cells, total CD4 T cells, total CD8 T cells, and NK cells. It is tempting to speculate that CD20^dim^ CD8^+^ T cells before treatment could be used to identify patients experiencing early disease activity after initial anti-CD20 therapy with the potential to offer these patients a modified personalized treatment. However, this would first require validation studies with larger cohorts, especially with more patients suffering from an early relapse after anti-CD20 treatment.

The following lessons can be learned from this study:

1) By analyzing blood immune profiles of patients receiving disease-modifying immune therapies, we can not only see and learn how therapy impacts the immune system (“mode of action” type of investigations) but also gain a deeper understanding of the underlying pathogenesis in a reverse translational approach. Those approaches will become even more powerful by incorporating novel single-cell omic technologies and multiple compartments (22). 2) CD20^dim^ T cells, particularly CD20^dim^ CD8^+^ T cells, may leave the circulation into the CNS already before the start of anti-CD20 therapy and contribute to relapse biology. This finding implies that anti-CD20 treatment is a highly effective therapy, but already-ongoing relapse biology cannot be modified, a notion that basically all used disease-modifying treatments share, even fast-acting leukocyte trafficking-inhibition agents. Moreover, those findings imply that CD20-expressing T cells should be depleted by a further dose of anti-CD20 therapy. Therapy should therefore not be discontinued too early because an insufficient effect is mistakenly assumed. 3) Early repopulating B cells exhibit a transitional anti-inflammatory phenotype and are probably not related to early MS disease activity after anti-CD20 therapy. However, effects would be expected to be reversible because B cell depletion therapy is not a reconstitution therapy in the sense that it fully restores a new immune system (23).

" The authors provide an in vivo study of anti-CD20 treatment on both T and B cells and their interactions, thereby acknowledging that MS is an immune-regulatory network disorder, not a disease of a single cell subset."

Consequently, proinflammatory memory B cells might reappear in the absence of continuous B cell depletion. 4) Frequency of pretreatment CD20^dim^ CD8^+^ T cells may have the potential in predicting early disease activity after anti-CD20 therapy. Yet, this would clearly necessitate larger multicenter biomarker studies in well-curated, longitudinal cohorts. In the near future, similar approaches will hopefully make personalized neuroimmunology not only wishful thinking but also practical reality.
